# Adult Wilms Tumour: A Case Report of an Atypical Presentation and Literature Review

**DOI:** 10.7759/cureus.101238

**Published:** 2026-01-10

**Authors:** Miguel Gil, José Cabrita Carneiro, João Cabral Pimentel, Ana Meireles, Luís Campos Pinheiro

**Affiliations:** 1 Department of Urology, Unidade Local de Saúde de São José, Lisbon, PRT; 2 Department of Anatomical Pathology, Unidade Local de Saúde de São José, Lisbon, PRT

**Keywords:** adult nephroblastoma, adult wilms tumour, multimodal therapy, risk-adapted treatment, risk stratification

## Abstract

Adult Wilms tumour is a rare renal malignancy. Although it shares histopathological features with its paediatric counterpart, its genetic landscape and underlying biological behaviour remain incompletely understood. Owing to its rarity, staging and treatment strategies in adults are largely extrapolated from paediatric clinical trials. The adoption of these protocols in adult practice has led to a significant improvement in outcomes over recent decades. Current management relies on a multimodal, risk-adapted approach, incorporating surgery, chemotherapy, and, when indicated, radiotherapy. We present the case of a 30-year-old woman diagnosed with stage I adult Wilms tumour following an atypical clinical presentation. This case underscores the diagnostic and therapeutic challenges associated with Wilms tumour in the adult population and highlights the importance of a multidisciplinary approach to management.

## Introduction

Kidney cancer ranks as the 14th most commonly diagnosed malignancy and the 16th leading cause of cancer-related mortality worldwide, accounting for approximately 434,000 new cases and 156,000 deaths each year [1]. It has a 1.5:1 male predominance and a peak incidence in the seventh decade of life [2]. Risk factors include genetic determinants and modifiable lifestyle-associated risk factors, such as smoking, obesity, hypertension, and occupational exposure [3]. Currently, only about 30% of patients are diagnosed after symptom-driven evaluation, whereas most cases are identified incidentally on imaging [2]. Kidney cancer comprises a heterogeneous group of tumours. In the most recent World Health Organization classification of renal tumours, renal cell tumours constitute the vast majority, whereas metanephric tumours, mixed epithelial and stromal tumours, renal mesenchymal tumours, embryonal neoplasms, and miscellaneous renal tumours are rare [4]. Wilms tumour (nephroblastoma) is a rare malignancy classified among the embryonal neoplasms of the kidney.

Wilms tumour is the most common childhood renal malignancy, accounting for 6% of all paediatric cancers [5]. The median age at diagnosis is between 3 and 4 years, and 90% of cases are diagnosed in children younger than 7 years [6]. The most common presentation is a painless abdominal mass [7]. However, clinical presentation may include abdominal pain, anorexia, vomiting, malaise, hypertension (25%), congenital anomalies (13-28%), haematuria (up to 30%), and coagulopathy (up to 10%) [5].

Most tumours are sporadic and unilateral (90%), whereas 10% are bilateral or multifocal, likely resulting from a germline mutation that confers genetic predisposition, and presenting at an earlier age [8,9]. Conditions associated with an increased risk of Wilms tumour include WT1-associated syndromes, familial Wilms tumour, childhood overgrowth syndromes, tumour predisposition syndromes and constitutional chromosomal abnormalities [10]. In up to 15% of patients, Wilms tumour arises in the context of a predisposition syndrome or a germline mutation [8]. However, true familial Wilms tumour is exceptionally rare (<1% of cases), with most instances resulting from de novo germline mutations rather than hereditary transmission [9]. Wilms tumour derives from pluripotent embryonic renal precursor cells that differentiate into the three hallmark histological components: blastemal, epithelial and stromal [9].

In contrast to its paediatric counterpart, adult Wilms tumour is exceedingly rare, representing less than 1% of all renal neoplasms [11]. Median age at diagnosis is 34 years, with an estimated incidence in Europe of 0.19 per million [6]. Typical clinical presentation in adults includes pain and haematuria [12]. Despite the absence of histopathological differences between adult and childhood Wilms tumour, the genetic landscape and predisposing conditions in adult cases remain poorly understood due to the paucity of studies. Moreover, there are no standardised protocols for its management, and current knowledge relies primarily on case reports and on experience derived from paediatric patients. Consequently, adult Wilms tumour represents a diagnostic and therapeutic challenge and has historically been associated with a poorer prognosis. We report a rare case of adult Wilms tumour with an atypical presentation, emphasising the diagnostic challenges and implications for clinical management.

## Case presentation

**Figure 1:**
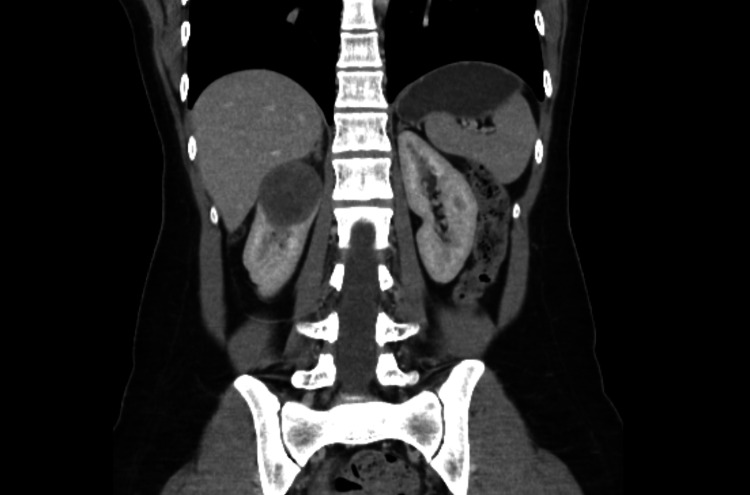
Coronal thoracoabdominopelvic CT showing a 45 x 40 mm complex cystic lesion consistent with a haemorrhagic cyst CT: computed tomography

A 27-year-old female patient was referred to the Urology Department for evaluation and follow-up of a complex cystic renal lesion. She was previously healthy and a nonsmoker. Three months earlier, she had attended the emergency department of another institution with right flank pain and fever. Laboratory tests revealed elevated inflammatory markers. Radiological assessment with computed tomography (CT), followed by magnetic resonance imaging (MRI), identified a 45 x 40 mm complex cystic lesion with internal septation in the upper pole of the right kidney, consistent with a haemorrhagic cyst (Figure 1). Her symptoms resolved with antibiotic therapy.

**Figure 2:**
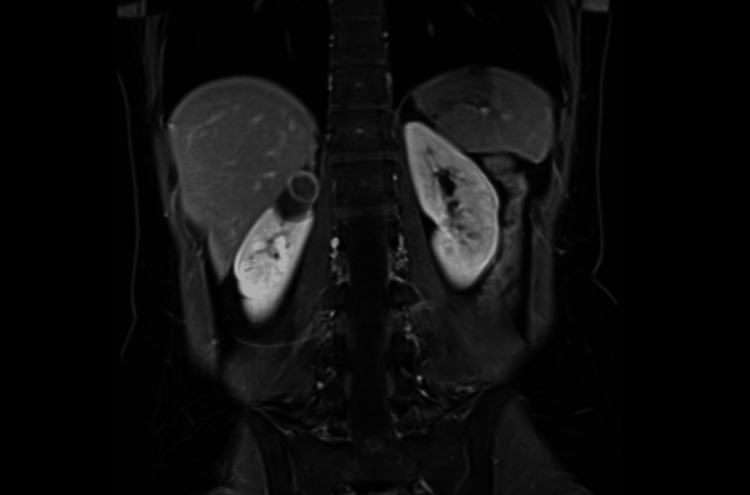
Coronal T1-weighted MRI showing a bilocular 35 x 24 mm lesion consistent with an involuting haemorrhagic cyst, classified as Bosniak IIF

**Figure 3:**
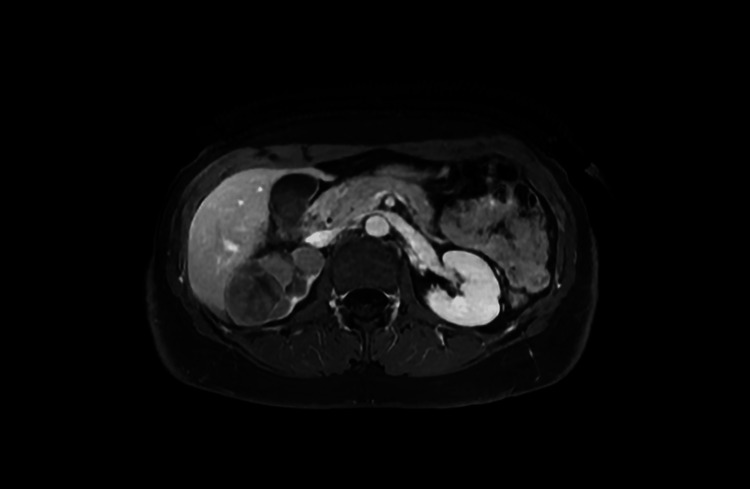
Axial contrast-enhanced T1-weighted MRI showing a 63 x 40 mm predominantly solid lesion, suggestive of primary renal malignancy

A follow-up scheme was implemented with alternating MRI and ultrasound, showing a gradual and unequivocal reduction of the cystic renal mass, which consistently exhibited features of an involuting haemorrhagic cyst, with a 3 mm thick wall in the upper portion, no nodular component and no enhancement, thus fulfilling the criteria for a Bosniak IIF lesion (Figure 2). The lesion eventually reached its smallest dimensions at the 20- and 29-month follow-up evaluations, measuring 16 mm and 21 mm, respectively. She was subsequently lost to follow-up for more than one year and returned 49 months after the initial episode, at the age of 30, with both ultrasound and MRI revealing a predominantly solid lesion measuring 63 x 40 x 37 mm, containing small internal cystic areas, showing contrast enhancement, and highly suggestive of a primary renal malignancy (Figure 3). Systemic staging with thoracic, abdominal and pelvic CT confirmed the absence of lymph nodes or distant metastases. The only additional finding was a small 4 mm nodular lesion in the right ovary, consistent with a small teratoma.

**Figure 4:**
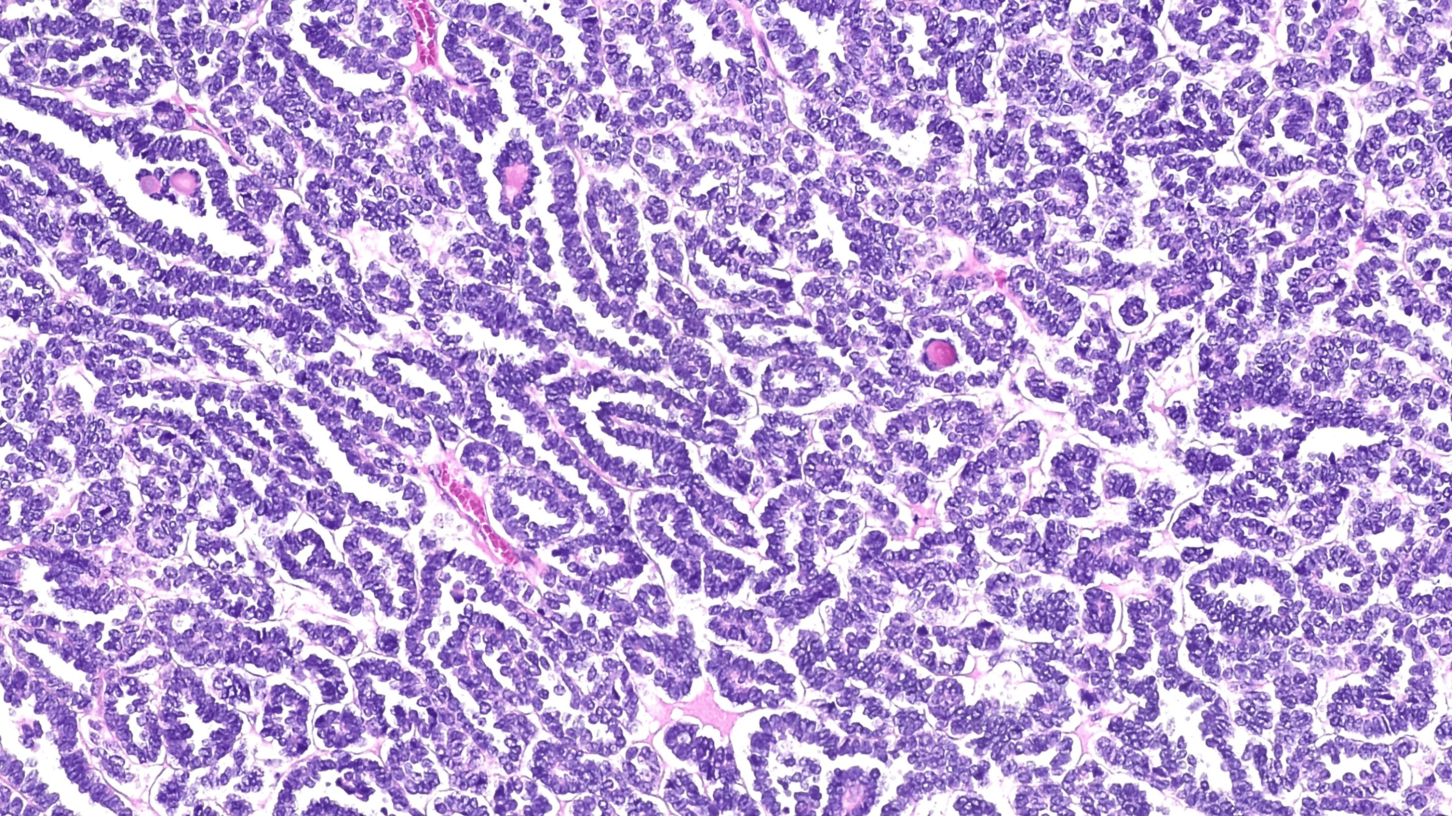
Epithelial components with mostly tubular and glomeruloid structures. Some microcalcifications are seen (H&E). Magnitude: 100x H&E: hematoxylin and eosin

**Figure 5:**
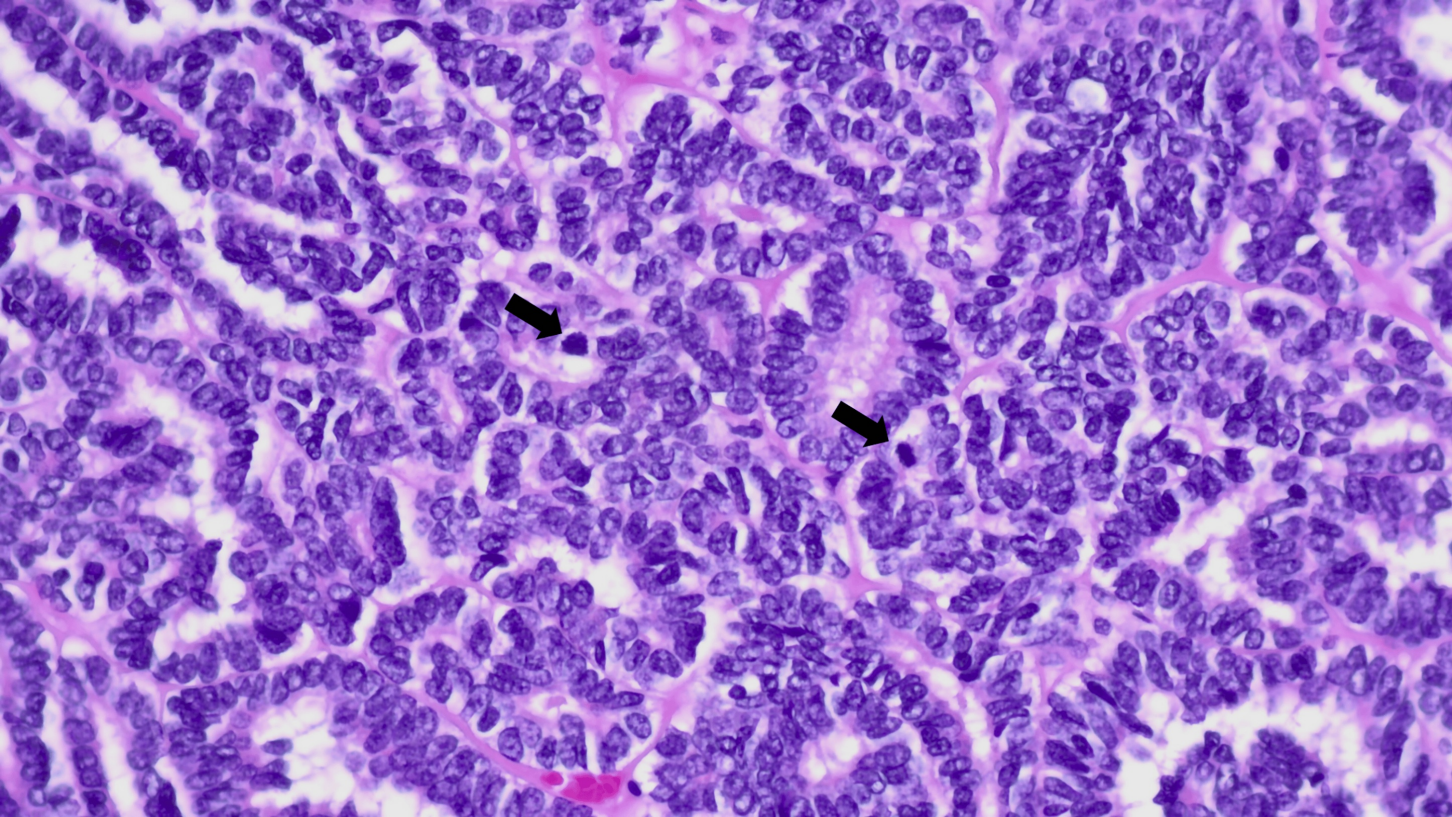
The neoplastic cells are basaloid with scant cytoplasm and mostly round to ovoid nuclei with membrane irregularity. No anaplastic features were seen. There are visible mitotic figures (black arrows). H&E. Magnitude: 400x H&E: hematoxylin and eosin

**Figure 6:**
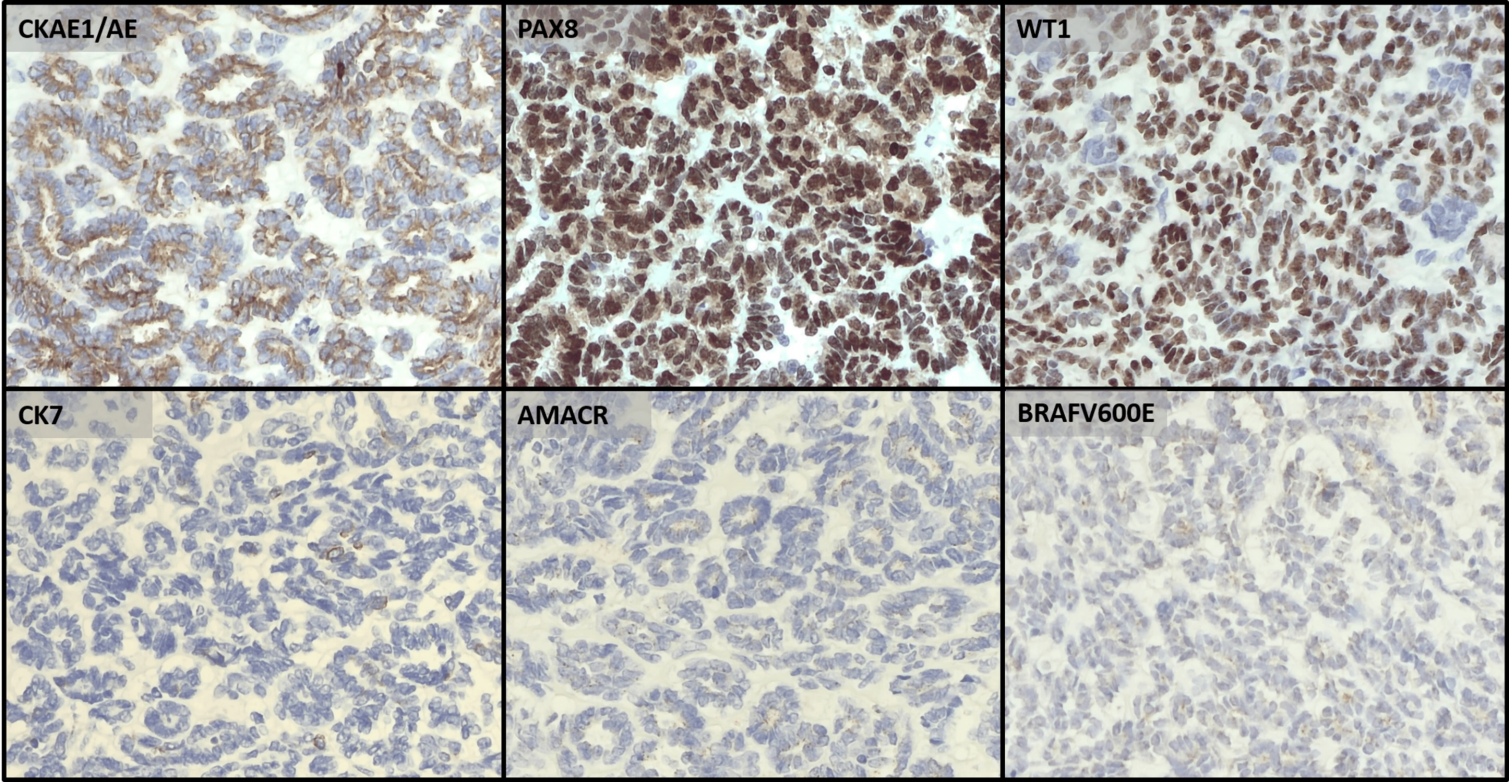
The neoplastic cells are positive for CKAE1/AE3, PAX8 and WT1 and negative for CK7, AMACR and BRAF V600E See text for complete immunohistochemical profile. Magnitude: 200x

She underwent laparoscopic transperitoneal right radical nephrectomy. No intraoperative or postoperative complications were recorded. Histological evaluation revealed a multinodular neoplasm with thick collagenous septa and tubular and focally glomeruloid patterns (Figure 4). The neoplastic cells were basaloid with round to oval nuclei and scant cytoplasm and mild nuclear pleomorphism. Numerous mitotic figures and areas of tumour necrosis were observed (Figure 5). There was no evidence of anaplasia or atypical mitoses. The neoplasia expressed PAX8, WT1, CD56, CD57, and focal EMA and was negative for CK7, alpha-methylacyl CoA racemase, GATA3, TTF1, carbonic anhydrase IX, and ALK1. Focal staining for BRAF V600E was noted but considered negative (Figure 6). INI1 expression was retained. Subsequent PCR-based molecular testing confirmed the absence of a BRAF V600E mutation. The morphological features, in conjunction with the immunohistochemical profile, support the diagnosis of a nephroblastoma with a predominant epithelial component and no anaplasia. The tumour was confined to the kidney, and surgical margins were negative. No nephrogenic rests were identified in the adjacent parenchyma.

Subsequently, the patient was started on adjuvant chemotherapy with the 18-week EE-4A regimen, comprising vincristine and actinomycin D. Follow-up thoracoabdominopelvic CT performed three months post-operatively revealed no evidence of recurrence. The patient remains asymptomatic to date

## Discussion

This case report presents several distinctive features. Firstly, the atypical evolution of an apparently benign lesion that had shown unequivocal and progressive reduction in size over time, followed by an unexpected and significant increase with clearly malignant characteristics, is difficult to explain accurately in light of current medical understanding. The most likely hypothesis is that the acute presentation with flank pain and fever resulted from a haemorrhagic episode complicated by secondary infection of the cystic lesion. However, it is impossible to determine with certainty whether the tumour was already present at that time. If it was, the reason why malignant features only became apparent four years later remains unclear. This case underscores the fundamental importance of careful follow-up in Bosniak IIF lesions.

Given the rarity of adult Wilms tumour and the absence of large-scale studies, current knowledge is extrapolated from management in paediatric patients. Consequently, several aspects of paediatric practice warrant consideration.

Wilms tumour management is based on collaborative trials conducted by large multi-institutional cooperatives, among which the leading international groups are the European Société Internationale d’Oncologie Pédiatrique (SIOP) and the North American National Wilms Tumor Study/Children’s Oncology Group (COG). These groups have developed two distinct strategies that have been evaluated and are currently established in clinical practice [8]. In Europe, most children receive preoperative chemotherapy followed by surgery, according to the SIOP protocol. In contrast, in North America, children traditionally undergo immediate nephrectomy followed by chemotherapy, following the COG strategy. The main ongoing trials recruiting patients include the UMBRELLA SIOP-RTSG 2016 study in Europe and the AREN2231 trial in North America [13,14].

At diagnosis, the tumour is staged as localised, metastatic or bilateral. However, definitive staging is only possible after nephrectomy. According to the SIOP protocol, staging is defined following surgery after preoperative chemotherapy, whereas in the COG pathway, staging is established after initial surgery [15]. However, both staging systems are similar [16]. In stage I, the tumour is confined to the kidney. In stage II, the tumour is not confined to the kidney (i.e. invasion into the renal capsule or into perirenal fat) or may infiltrate the vena cava but is completely resected with negative surgical margins. In stage III, there is residual disease confined to the abdomen, including positive surgical margins, tumour rupture, peritoneal contamination, or lymph node involvement. In stage IV, there are haematogenous metastases or positive lymph nodes outside the abdominopelvic region. In stage V, there are bilateral renal tumours at diagnosis.

Risk stratification is essential not only for defining prognosis but also for guiding treatment. The two most significant prognostic factors are stage and histology [15]. The SIOP classification stratifies tumours into low-, intermediate-, and high-risk groups based on the extent of chemotherapy-induced changes, the relative proportions of the different viable Wilms tumour components, and the presence of anaplasia [17]. Consequently, low-risk tumours comprise completely necrotic Wilms tumours owing to preoperative treatment, intermediate-risk tumours include epithelial-type, stromal-type, mixed-type, and regressive-type tumours as well as Wilms tumours with focal anaplasia, and high-risk tumours include blastemal-type and diffuse anaplasia [17]. Moreover, in stage II and III intermediate-risk non-stromal and non-epithelial types, a tumour volume of ≥500 mL is associated with a poorer outcome and warrants more aggressive treatment [13]. Similarly to SIOP, in COG trials, risk stratification was traditionally based on stage and histology. However, COG has evolved to integrate age, tumour molecular analysis (loss of heterozygosity of 1p, 16q and 11p15, and 1q gain), response of pulmonary metastases to chemotherapy and extrapulmonary metastases into a risk stratification model [14].

Wilms tumour is both chemosensitive and radiosensitive, and multimodal therapy has led to a significant improvement in survival from 30% in the 1930s to over 85% nowadays [5]. For localised disease, the current SIOP protocol recommends preoperative chemotherapy with actinomycin D and vincristine before surgery, allowing tumour downstaging, reduced use of postoperative radiotherapy and doxorubicin, and a personalised assessment of tumour chemosensitivity [13]. Postoperative treatment is determined by tumour stage and the aforementioned histological risk stratification [13]. For stage I, low-risk tumours require no adjuvant therapy, whereas intermediate-risk tumours are treated with four weeks of actinomycin D and vincristine, and high-risk tumours with 27 weeks of actinomycin D, vincristine, and doxorubicin. In stages II and III, low- and intermediate-risk tumours require 27 weeks of adjuvant actinomycin D and vincristine, except for non-stromal and non-epithelial intermediate-risk tumours with a tumour volume ≥500 mL, which also require additional doxorubicin. High-risk tumours are treated with a 34-week chemotherapy regimen comprising etoposide, carboplatin, cyclophosphamide and doxorubicin. Additionally, stage II high-risk tumours with diffuse anaplasia, as well as all stage III intermediate- and high-risk tumours, receive additional flank radiotherapy. For metastatic disease, SIOP recommends a combination of actinomycin D, vincristine and doxorubicin for six weeks, followed by imaging reassessment of local and distant staging. With this regimen, approximately two-thirds of patients achieve a complete metastatic response, and subsequent treatment decisions are made [13].

The treatment guidelines in the current COG protocol also support a risk-stratified multimodal treatment algorithm, adapted to the COG nephrectomy-first strategy [14]. Notably, in an attempt to prevent treatment-related toxicity, deintensification strategies with no adjuvant chemotherapy after nephrectomy have recently been evaluated for very-low-risk patients. Consequently, patients with stage I Wilms tumour, with normal molecular features, epithelial histology and no Wilms tumour-predisposing conditions may be offered nephrectomy only, with a four-year event-free survival rate of 96.2% and overall survival rate of 100% [14,18].

Regarding the surgical resection of the primary tumour, a few considerations are noteworthy. Radical nephrectomy remains the mainstay, while partial nephrectomy is generally discouraged. However, it may be considered in specific circumstances, such as small tumour volume, single functioning kidney or synchronous bilateral tumours. Nevertheless, tumour spillage must be avoided due to the increased risk of local recurrence, and lymph node sampling is crucial for accurate staging.

Adult Wilms tumour poses several clinical challenges. Radiological appearance of Wilms tumour is indistinguishable from other adult renal malignancies. Consequently, the diagnosis of Wilms tumour in adults is often unexpected, typically being established after nephrectomy performed for a presumed renal cell carcinoma, and thus precluding the possibility of receiving preoperative chemotherapy. Moreover, the usual delay in pathology review prevents the initiation of adjuvant therapy within the recommended 7-14 days after nephrectomy [11]. The diagnosis of adult Wilms tumour is usually made following the criteria proposed by Kilton et al. which include (1) primary renal neoplasm, (2) primitive blastematous spindle or round cell component, (3) formation of abortive or embryonal tubular or glomeruloid structures, (4) no area of tumour diagnostic of renal cell carcinoma, (5) histopathological confirmation, and (6) age > 15 years [19].

Historically, adult Wilms tumour was associated with a worse prognosis than paediatric Wilms tumour. However, recent data have shown an improved outcome when adult patients are timely treated following the therapeutic paediatric protocols. This improvement has resulted in a marked increase in survival, with a three-year overall survival of 24% (compared with 74% in children) reported in 1982, rising to 67% in 1990, and a five-year overall survival of 83% in 2004 (43% of patients had advanced-stage disease) [11].

The chemotherapy regimen with only actinomycin D and vincristine should be reserved for stage I Wilms tumours without anaplasia, with negative lymph node sampling, and in which chemotherapy can be started within 30 days of nephrectomy [11]. In the remaining patients with non-anaplastic Wilms tumours, doxorubicin should be included, whereas a chemotherapy regimen comprising carboplatin, cyclophosphamide, etoposide, and doxorubicin should be given for anaplastic tumours irrespective of stage [13]. Unlike paediatric patients, radiotherapy is indicated in all adult stage II Wilms tumour [13]. Due to the exceptional rarity of adult Wilms tumour, review of histopathological findings by expert uropathologists, coupled with management planning within multidisciplinary team boards, is of critical importance.

## Conclusions

Adult Wilms tumour is an exceedingly rare malignancy, with a challenging diagnosis that often causes delays in starting adjuvant treatment. Current knowledge is largely extrapolated from paediatric trials, but management should be tailored to the specific features of adult Wilms tumour. A multimodal, risk-adapted, multidisciplinary treatment strategy is essential to ensure optimal care, maximising oncological survival while minimising long-term toxicities. This case highlights the diagnostic complexity and the importance of prompt recognition to guide management. Further adult-focused data and collaborative research initiatives are needed to refine management strategies and improve outcomes in this rare population.

## References

[1] Bray F, Laversanne M, Sung H, Ferlay J, Siegel RL, Soerjomataram I, Jemal A (2024). Global cancer statistics 2022: GLOBOCAN estimates of incidence and mortality worldwide for 36 cancers in 185 countries. CA Cancer J Clin.

[2] Capitanio U, Montorsi F (2016). Renal cancer. Lancet.

[3] Cirillo L, Innocenti S, Becherucci F (2024). Global epidemiology of kidney cancer. Nephrol Dial Transplant.

[4] Moch H, Amin MB, Berney DM (2022). The 2022 World Health Organization Classification of tumours of the urinary system and male genital organs-part A: renal, penile, and testicular tumours. Eur Urol.

[5] Kalapurakal JA, Dome JS, Perlman EJ, Malogolowkin M, Haase GM, Grundy P, Coppes MJ (2004). Management of Wilms’ tumour: current practice and future goals. Lancet Oncol.

[6] Mitry E, Ciccolallo L, Coleman MP, Gatta G, Pritchard-Jones K, Eurocare Working Group (2006). Incidence of and survival from Wilms' tumour in adults in Europe: data from the EUROCARE study. Eur J Cancer.

[7] Dumba M, Jawad N, McHugh K (2015). Neuroblastoma and nephroblastoma: a radiological review. Cancer Imaging.

[8] Treger TD, Chowdhury T, Pritchard-Jones K, Behjati S (2019). The genetic changes of Wilms tumour. Nat Rev Nephrol.

[9] Rivera MN, Haber DA (2005). Wilms' tumour: connecting tumorigenesis and organ development in the kidney. Nat Rev Cancer.

[10] Scott RH, Stiller CA, Walker L, Rahman N (2006). Syndromes and constitutional chromosomal abnormalities associated with Wilms tumour. J Med Genet.

[11] Segers H, van den Heuvel-Eibrink MM, Pritchard-Jones K (2011). Management of adults with Wilms' tumor: recommendations based on international consensus. Expert Rev Anticancer Ther.

[12] Huszno J, Starzyczny-Słota D, Jaworska M, Nowara E (2013). Adult Wilms' tumor-diagnosis and current therapy. Cent European J Urol.

[13] van den Heuvel-Eibrink MM, Hol JA, Pritchard-Jones K (2017). Position paper: Rationale for the treatment of Wilms tumour in the UMBRELLA SIOP-RTSG 2016 protocol. Nat Rev Urol.

[14] Benedetti DJ, Cost NG, Ehrlich PF (2025). Updated favourable-histology Wilms tumour risk stratification: rationale for future Children's Oncology Group clinical trials. Nat Rev Urol.

[15] Kaste SC, Dome JS, Babyn PS (2008). Wilms tumour: prognostic factors, staging, therapy and late effects. Pediatr Radiol.

[16] Ahmed HU, Arya M, Tsiouris A, Sellaturay SV, Shergill IS, Duffy PG, Mushtaq I (2007). An update on the management of Wilms' tumour. Eur J Surg Oncol.

[17] Vujanić GM, Gessler M, Ooms AH (2018). The UMBRELLA SIOP-RTSG 2016 Wilms tumour pathology and molecular biology protocol. Nat Rev Urol.

[18] Parsons LN, Mullen EA, Geller JI (2020). Outcome analysis of stage I epithelial-predominant favorable-histology Wilms tumors: a report from Children's Oncology Group study AREN03B2. Cancer.

[19] Kilton L, Matthews MJ, Cohen MH (1980). Adult Wilms tumor: a report of prolonged survival and review of literature. J Urol.

